# Avaliação Não Invasiva da Pressão Arterial Central e da Forma de Onda Intracraniana em Pacientes Hipertensos: Um Estudo Transversal

**DOI:** 10.36660/abc.20240778

**Published:** 2025-04-24

**Authors:** Sayuri Inuzuka, Mikaelle Costa Correia, Matheus Martins da Costa, Thiago Oliveira Costa, Priscila Valverde de Oliveria Vitorino, Polyana Vulcano de Toledo Piza, Gustavo Frigieri, Ana Luiza Lima Sousa, Antonio Coca, Weimar Kunz Sebba Barroso

**Affiliations:** 1 Unidade de Hipertensão Arterial Universidade Federal de Goiás Goiânia GO Brasil Unidade de Hipertensão Arterial – Universidade Federal de Goiás, Goiânia, GO – Brasil; 2 Pontifícia Universidade Católica de Goiás Escola de Ciências Sociais e da Saúde Goiânia GO Brasil Pontifícia Universidade Católica de Goiás – Escola de Ciências Sociais e da Saúde, Goiânia, GO – Brasil; 3 Hospital Israelita Albert Einstein Goiânia GO Brasil Hospital Israelita Albert Einstein, Goiânia, GO – Brasil; 4 Brain4care São Paulo SP Brasil Brain4care, São Paulo, SP – Brasil; 5 Laboratório de investigação médica 62 Universidade de São Paulo São Paulo SP Brasil Laboratório de investigação médica 62, Universidade de São Paulo, São Paulo, SP – Brasil; 6 School of Health and Life Sciences Universitat Abat Oliba CEU CEU Universities Barcelona Espanha School of Health and Life Sciences. Universitat Abat Oliba CEU, CEU Universities, Barcelona – Espanha; 7 Hospital das Clínicas da Universidade Federal de Goiás Goiânia GO Brasil Hospital das Clínicas da Universidade Federal de Goiás, EBSERH, Goiânia, GO – Brasil

**Keywords:** Acidente Vascular Cerebral, Transtornos Cerebrovasculares, Disfunção Cognitiva, Hipertensão, Pressão Intracraniana

## Abstract

**Fundamento:**

Há uma forte associação entre hipertensão e doença cerebrovascular, principalmente acidente vascular cerebral e déficit cognitivo. Porém, os mecanismos dessa relação não são completamente compreendidos.

**Objetivos:**

Analisar a relação da pressão arterial periférica, pressão arterial central e da rigidez arterial, com a pressão intracraniana (PIC) em pacientes com hipertensão crônica.

**Métodos:**

Indivíduos adultos foram consecutivamente incluídos no estudo entre novembro de 2022 e agosto de 2023. O ponto de corte identificado para definir hipertensão intracraniana (HTIC) pela razão de onda de pico (P2/P1) foi 1,2, e o ponto de corte para o tempo para o pico (TPP) foi 0,25. O nível de significância adotado na análise estatística foi 5%.

**Resultados:**

Um total de 145 pacientes (32 homens e 113 mulheres) com hipertensão crônica (média de tempo desde o diagnóstico de 20 ± 12 anos) foram avaliados por um período de 10 meses. A idade mediana foi 69,0 (61,8 – 75,7) anos e o índice de massa corporal mediano foi 29,0 (25,4 – 33,1) Kg/m^2^. O valor mediano da razão P2/P1 para todas as cortes foi 1,4 (1,2 – 1,5) e do TPP 0,24 (0,21 – 0,29). A análise foi realizada considerando presença ou não de HTIC, e parâmetros da pressão arterial central e da velocidade de onda de pulso. Observou-se valores mais altos de pressão arterial sistólica (PAS), pressão arterial diastólica (PAD), e PAD periférica entre pacientes com HTIC com base na razão P2/P1.

**Conclusões:**

Os níveis de PAS central estão mais relacionados com HTIC que valores de PAS periférica medidos no consultório, não sendo observada tal diferença na PAD. Esse achado levanta a questão do método mais adequado de avaliação da pressão arterial em pacientes hipertensos com lesão cerebral.

## Graphical abstract

**Figura Central:**
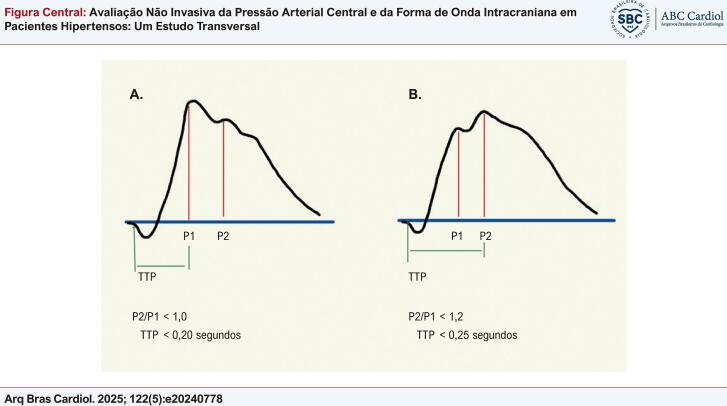
Avaliação Não Invasiva da Pressão Arterial Central e da Forma de Onda Intracraniana em Pacientes Hipertensos: Um Estudo Transversal Monitoramento em tempo real não invasivo por b4c da forma de onda da Pressão Intracraniana. Todos os dados coletados são imediatamente processados pelo programa analítico b4c, resultando em registros quantitativos e qualitativos. A formas de onda apresentando padrões de normalidade [P2/P1 < 1,1 e tempo para o pico (TPP) < 0,20 segundos] e B: padrões alterados (P2/P1 ≥ 1,2 e TPP ≥ 0,25 segundos).^7,8^

## Introdução

A hipertensão (HT) ainda é a principal causa de morte no mundo, e as doenças cerebrais, como acidente vascular cerebral (AVC) e déficit cognitivo, seguem como importantes problemas de saúde, afetando um em cada cinco americanos com idade igual ou superior a 65 anos.^1,2^

Existe uma forte associação entre HT e doenças cerebrovasculares. Porém, os mecanismos dessa relação ainda não são completamente compreendidos, necessitando de mais estudos. Já está bem estabelecido que a elevação da pressão arterial (PA) pode causar lesão estrutural e funcional no leito arterial; mas em que nível esse processo se inicia e a que extensão ele afeta o cérebro ainda precisa ser elucidado.^2-5^

Um estudo recente avaliando a forma de onda da pressão intracraniana (PIC) de maneira não invasiva em pacientes com hipertensão crônica encontrou uma prevalência de 45,6% de hipertensão intracraniana (HTIC), levantando algumas questões sobre a real capacidade de proteção da autorregulação cerebral e da barreira cerebral vascular nessa população.^6-8^

Recentemente, as consequências da hemodinâmica arterial pulsátil, incluindo valores mais altos e PA e rigidez arterial, emergiram como um dos importantes mecanismos que afetam a função e a estrutura do coração. A presença de pulsatilidade aumentada nas pequenas artérias cerebrais, bem como o estiramento mecânico cíclico endotelial no cérebro tem o potencial de produzir depósitos de amiloide e reforça o papel não só do comportamento da PA periférica como também da PA central na saúde cerebral.^9,10^

A autorregulação hemodinâmica tem a capacidade de proteger o tecido e os vasos cerebrais, mas essa proteção pode estar alterada quando a hipertensão em longo prazo modifica a capacidade dos vasos cerebrais de adaptarem o fluxo ao aumento da PA. Nossa hipótese é a de que, após uma exposição em longo prazo à HT, o mecanismo protetor vascular do cérebro esteja deficiente. Por isso, desenvolvemos um estudo transversal para explorar a relação da PA central e periférica e da rigidez arterial com a PIC em pacientes com hipertensão crônica.

## Materiais e métodos

### Seleção dos pacientes

Uma coorte de pacientes adultos consecutivos com HT, atendidos no Centro de Pesquisas de Doenças Cardiometabólicas da Unidade de Hipertensão, Universidade Federal de Goiás, entre novembro de 2022 e agosto de 2023, foi incluída na análise transversal. O estudo foi conduzido de acordo com a resolução 466/2012 e foi aprovado pelo comitê de ética (70448823.1.0000.5078).

Após exame físico e avaliação de rotina da história médica, os seguintes parâmetros foram registrados: idade, sexo, índice de massa corporal (IMC) em Kg/m^2^, pressão arterial sistólica e pressão arterial diastólica periféricas (PASp e PADp, respectivamente) (mmHg) de consultório, PAS central (PASc) e PAD central (PADc) (mmHg), velocidade de onda de pulso (VOP) em m/s, índice de aumento (IA) (%), história de fatores de risco cardiovascular (CV) e doença CV – infarto do miocárdio (IM), AVC, Diabetes Mellitus tipo 2 (DM2), e dislipidemia, bem como o número e a classe de medicamentos anti-hipertensivos. Todas essas variáveis foram coletadas no mesmo dia em que a forma de onda da PIC foi medida e foram organizadas usando a ferramenta de captura de dados eletrônicos REDCap.

### Avaliação da pressão arterial periférica e central

Os seguintes parâmetros: PASp, PADp, PASc, PADc, VOP e IA foram obtidos usando um dispositivo oscilométrico, o Mobil-O-Graph® (IEM, Stolberg, Alemanha),^11,12^ com base em medidas em triplicata da VOP com calibração C1 (média diastólica). Os dados foram processados com o algoritmo ARCSolver® (*Austrian Institute of Technology*, Viena, Áustria). As medidas foram tomadas no braço esquerdo, com o paciente sentado com as pernas descruzadas, os pés com a planta inteira no chão, e o braço em repouso, apoiado sobre a mesa, ao nível do coração. Os pacientes foram orientados a evitar a ingestão de bebida alcoólica por 10 horas e de cafeína, tabagismo, e exercício por três horas antes das medidas, e descansarem por 10 minutos antes do procedimento. Foram obtidas três leituras da PA, e calculada a média das três medidas.^13,14^

### Avaliação da pressão intracraniana

Uma medida não invasiva da PIC foi realizada com os pacientes deitados, e monitorados por sete minutos. O primeiro e o último minuto foram descartados. O sensor validado brain4care (b4c)^8,15^ foi posicionado na cabeça dos pacientes, e a morfologia das ondas adquiridas por um sensor de deformação que poderia detectar e monitorar deformações nanométricas do crânio durante cada ciclo cardíaco.

Pelas medidas tradicionais invasivas, a HTIC foi definida como PIC sustentada (>5 minutos) por mais de 20 mmHg.^16^ Utilizando a avaliação não invasiva b4c, o ponto de corte identificado para definir HTIC pela razão P2/P1 foi ≥ 1,2, e o ponto de corte para o tempo para o pico (TPP) foi ≥ 0,25 segundos. A razão P2/P1 refere-se à razão entre a amplitude P1 (resultante da transmissão do fluxo sanguíneo cerebral sistólico) e a amplitude P2 (associada com complacência cerebral à PIC); e o TPP é definido como o tempo (em segundos) desde o início da forma de onda até P1. Os valores de P2/P1 entre 1,0 e 1,19, e os valores de TPP entre 0,20 e 0,24 foram considerados uma zona cinza de complacência intracraniana anormal, mas não HTIC^17-19^ (Figura Central).

### Análise estatística

Os dados foram registrados na plataforma REDCap e analisados com o programa Jamovi, versão 2.3.28. As variáveis categóricas foram apresentadas em frequências e proporções. As variáveis quantitativas foram primeiramente analisadas em termos de distribuição, aplicando-se o teste de Shapiro-Wilk; esses dados foram expressos em média ± desvio padrão (DP) ou mediana e intervalo interquartil, de acordo com a normalidade dos dados. Para comparar os valores de P2/P1 e de TPP entre as categorias das variáveis quantitativas, o teste de Mann-Whitney foi usado para análises bivariadas e, para as correlações, o teste de Spearman foi usado. Um nível de significância de p<0,05 foi adotado.

## Resultados

Uma coorte de 145 paciente foi incluída no estudo. O tempo médio desde o diagnóstico de hipertensão foi de 20,0 ± 12,0 anos. Variáveis clínicas, antropométricas e sociodemográficas estão apresentadas na Tabela 1. A comparação dos valores P2/P1 e TTP de acordo com os fatores de risco CVs, dados sociodemográficos e antropométricos não identificou diferenças, exceto pelo TPP entre homens e mulheres (Tabela 2).

**Tabela 1:** – Características sociodemográficas, índice de massa corporal, comorbidades, número de anti-hipertensivos, e parâmetros de pressão arterial central e de pressão intracraniana dos pacientes do estudo (n=145)

Variável	Média ± desvio padrão ou mediana (p25 – p75%) ou n (%)
**Idade (anos)**	69,0 (61,8 – 75,7)
**Sexo**	
Feminino	113 (77,9%)
Masculino	32 (22,1%)
**Índice de massa corporal (n=136)**	29,0 (25,4 – 33,1)
**Comorbidades**	
Diabetes	74 (51,0%)
Dislipidemia	129 (89,0%)
Infarto do miocárdio	20 (13,8%)
Acidente vascular cerebral	17 (11,7%)
**Número de anti-hipertensivos**	
1	35 (24,1%)
2-3	43 (29,7%)
≥ 3	57 (39,3%)
**Avaliação da pressão arterial**	
PASp (mmHg)	125,0 (115,0 – 139,0)
PADp (mmHg)	79,0 (72,0 – 86,0)
PASc (mmHg)	115,0 (160,0 – 129,0)
PADc (mmHg)	80,0 (73,0 – 87,0)
VOP (m/sec)	9,9 ± 1,9
IA (%)	25,0 (16,0 – 30,0)
Resistência vascular (s*mmHg/ml)	1,4 (1,3 – 1,5)
**Forma de onda da Pressão Intracraniana**	
P2/P1	1,4 (1,2 – 1,5)
TPP	0,24 (0,21 – 0,29)

PASp: pressão arterial sistólica periférica; PADp: pressão arterial diastólica periférica, PASc pressão arterial sistólica central; PADc: pressão arterial diastólica central; VOP: velocidade de onda de pulso; IA: índice de aumento; TPP: tempo para o pico. Goiânia, 2023.

Fonte: Os autores.

**Tabela 2:** – Comparação da relação dos picos (P2/P1) e do tempo para o pico (TPP) com os parâmetros clínicos, sociodemográficos e antropométricos (n=145)

	P2/P1		TPP	
Variável	Mediana (p25 – p75%)	p	Mediana (p25 – p75%)	p
**Idade**		0,949		0,143
< 65 anos	1,35 (1,23-1,52)		0,25 (0,23-0,29)	
≥ 65 anos	1,37 (1,17-1,52)		0,24 (0,20-0,28)	
**Sexo**		0,292		0,048
Feminino	1,37 (1,21-1,54)		0,25 (0,21-0,29)	
Masculino	1,33 (1,18-1,45)		0,23 (0,21-0,26)	
**Sobrepeso (BMI>25)**		0,929		0,225
Sim	1,36 (1,19-1,52)		0,24 (0,22-0,29)	
Não	1,36 (1,22-1,49)		0,23 (0,21-0,27)	
**Comorbidades**				
**Diabetes**		0,349		0,169
Sim	1,33 (1,20-1,49)		0,24 (0,21-0,27)	
Não	1,39 (1,19-1,57)		0,25 (0,21-0,29)	
**Dislipidemia**		0,334		0,614
Sim	1,35 (1,19-1,52)		0,24 (0,21-0,29)	
Não	1,43 (1,23-1,64)		0,26 (0,20-0,28)	
**Infarto do miocárdio**		0,744		0,234
Sim	1,34 (1,14-1,57)		0,22 (0,20-0,28)	
Não	1,36 (1,21-1,52)		0,25 (0,22-0,29)	
**Acidente vascular cerebral**		0,890		0,993
Sim	1,29 (1,15-1,58)		0,24 (0,20-0,29)	
Não	1,36 (1,20-1,52)		0,24 (0,21-0,28)	
**Número de anti-hipertensivos**		0,855		0,481
1	1,37 (1,22-1,52)		0,25 (0,22-0,28)	
2	1,27 (1,18-1,47)		0,24 (0,21-0,28)	
≥ 3	1,40 (1,22-1,53)		0,25 (0,21-0,29)	

P2/P1: razão entre a amplitude P1 (resultante da transmissão do fluxo sanguíneo cerebral sistólico) e a amplitude P2 (associada com complacência cerebral à pressão intracraniana); TPP: Tempo Para o Pico, isto é, tempo (em segundos) desde o início da forma de onda até P1; Teste de Mann-Whitney. Goiânia, 2023.

Fonte: Os autores.

Segundo resultados de estudos prévios com P2/P1, definimos P2/P1 <1,0 como normal, P2/P1 entre 1,0 e 1,19 como distúrbio da complacência intracraniana, e P2/P1 ≥ 1,2 como HTIC.^17-19^ A associação da HTIC com valores de PA central e VOP também foi avaliada. Valores mais altos de PASc, PADc e PADp foram encontrados nos pacientes com HTIC, identificados pelo critério da razão P2/P1. Nos pacientes com HTIC, identificados adotando-se o critério do TPP (≥0,25 segundos), tanto a PAD central como periférica foram mais altas nesses pacientes (Tabela 3). A Tabela 4 apresenta a análise de correlação das variáveis de PIC e medidas de PA central.

**Tabela 3:** – Análise bivariada de P2/P1 e do Tempo para o Pico (TPP) de acordo com os parâmetros da pressão arterial central e da velocidade de onda de pulso

	P2/P1			TTP		
	< 1,2	≥ 1,2	p	< 0,25	≥ 0,25	p
PASp	122,0 (112,0-133,0)	127,0 (116,0-139,0)	0,068	123,0 (115,0-136,0)	128,0 (115,5-140,5)	0,307
PADp	75,0 (70,0-81,0)	80,0 (74,0-86,0)	0,030	77,0 (71,0-82,8)	80,0 (75,0-88,0)	0,032
PASc	109,0 (103,0-117,0)	117,0 (107,8-131,0)	0,024	114,0 (106,0-123,0)	118,0 (107,0-131,0)	0,117
PADc	76,0 (71,0-82,0)	81,0 (75,0-88,0)	0,037	78,5 (72,0-84,0)	81,0 (76,0-89,0)	0,039
VOP	10,1±2,0	9,8±1,9	0,438	10,1±2,0	9,7±1,8	0,149
IA	25,0 (17,0-28,0)	25,0 (16,0-31,0)	0,572	25,0 (15,3-28,0)	26,0 (19,0-31,0)	0,190
RV	1,40 (1,30-1,50)	1,40 (1,30-1,53)	0,588	1,40 (1,30-1,54)	1,40 (1,30-1,50)	0,964

P2/P1: razão entre a amplitude P1 (resultante da transmissão do fluxo sanguíneo cerebral sistólico) e a amplitude P2 (associada com complacência cerebral à pressão intracraniana); TPP: tempo para o pico, isto é, tempo (em segundos) desde o início da forma de onda até P1; PASp: pressão arterial sistólica periférica; PADp: pressão arterial diastólica periférica, PASc pressão arterial sistólica central; PADc: pressão arterial diastólica central; VOP: velocidade de onda de pulso; IA: índice de aumento; RV: resistência vascular; Teste de Mann-Whitney.

Fonte: Os autores.

**Tabela 4:** – Análise de correlação da razão P2/P1 e do Tempo para o Pico (TPP) com parâmetros da velocidade de onda de pulso e da velocidade da onda de pulso (fonte: os autores)

	P2/P1		TPP	
	r	p	r	p
PASp	0,16	0,053	0,09	0,299
PADp	0,17	0,043	0,23	0,006
PASc	0,19	0,020	0,14	0,095
PADc	0,17	0,044	0,22	0,008
VOP	-0,02	0,767	-0,14	0,104
IA	0,06	0,501	0,12	0,157
RV	-0,01	0,889	-0,06	0,509

P2/P1: razão entre a amplitude P1 (resultante da transmissão do fluxo sanguíneo cerebral sistólico) e a amplitude P2 (associada com complacência cerebral à pressão intracraniana); TPP: tempo para o pico, isto é, tempo (em segundos) desde o início da forma de onda até P1; PASp: pressão arterial sistólica periférica; PADp: pressão arterial diastólica periférica, PASc: pressão arterial sistólica central; PADc: pressão arterial diastólica central; VOP: velocidade de onda de pulso; IA: índice de aumento; RV: resistência vascular; Teste de Mann-Whitney.

## Discussão

A coorte do estudo foi composta de pacientes com hipertensão essencial crônica com uma idade mediana de 69,0 (61,8 – 75,7) anos, uma alta prevalência de fatores de risco cardiometabólicos tais como DM2 (51,0%) e dislipidemia (89,0%), e doença CV prévia tais como IM (13,8%) e AVC (11,7%) (Tabela 1).

Embora a forte associação entre HT e doença cerebrovascular esteja bem estabelecida, especialmente AVC e déficit cognitivo, os mecanismos fisiopatológicos que envolvem dano cerebral induzido por hipertensão não são bem conhecidos. Parece que lesão cerebral secundária a distúrbios na barreira hematoencefálica e na autorregulação vascular ocorra antes da neurodegeneração, sendo importante um melhor entendimento dessa associação.^20-24^

Em um estudo prévio,^6^ encontramos uma prevalência inesperadamente alta (45,6%) de HTIP em pacientes hipertensos, principalmente entre as mulheres, apesar de não termos observados diferenças entre pacientes hipertensos controlados e não controlados por meio da avaliação da PA periférica. Esses achados levaram à hipótese de que, após uma exposição em longo prazo à HT, os mecanismos de proteção vascular do cérebro foram perdidos. Contudo, outra hipótese a ser considerada é a de que as medidas de PA periférica não sejam tão precisas para detectar tais diferenças, e talvez as medidas de PA central seriam mais sensíveis. A avaliação central não invasiva reflete valores da PA de grandes artérias, tais como aorta ou carótida e, por isso, é um marcador mais forte de morbidade e mortalidade CV que a PA periférica.^25,26^ Neste estudo, encontramos um valor mediano de 0,24 (0,21 – 0,29) segundos na população estudada. A Tabela 1 apresenta valores de PA central e periférica, e a análise da VOP nessa população hipertensa com alto risco, com uma alta prevalência de fatores de risco e doenças CVs, e uma VOP mediana próxima a 10 (9,9 ± 1,9) m/s, que caracteriza lesão vascular de acordo com estudos e diretrizes prévios.^14,27-29^

Ao analisar diferenças nos valores médios da razão P2/P1 e do TPP nas variáveis clínicas, antropométricas e sociodemográficas (Tabela 2), a única diferença observada foi um TPP mais longo entre as mulheres. Outros autores, bem como nosso grupo de pesquisa, discutiram anteriormente que uma exposição em longo prazo à PA elevada poderia ser responsável por dados importantes nos mecanismos neuroprotetores, e por uma perda da capacidade de se manter valores de ICP normais nessa população, mesmo quando a PA está bem controlada.^3,6,9,30^ Importante mencionar que a incidência de AVC e de demência é mais alta nas mulheres, e os resultados de um TPP mais longo, bem como uma razão P2/P1 mais alta entre as mulheres em nosso estudo, corrobora a hipótese de que a capacidade autorregulatória da PCI e a permeabilidade da barreira hematoencefálica sejam afetadas de maneira diferente em homens e mulheres.^9,31^

Muitos mecanismos fisiopatológicos foram associados a IMC e HTIC elevados, e a obesidade é reconhecida como um fator de risco para HTIC.^32,33^ Dados recentes sugerem a associação da HTIC com resistência insulínica, DM2 e doença CV.^34,35^No entanto, não encontramos nenhuma relação do sobrepeso, da razão P2/P1 ou do TPP com a presença de DM2 ou dislipidemia, talvez devido ao pequeno tamanho da nossa amostra. Finalmente, na análise considerando os pontos de corte de 1,2 e 0,25 segundos para P2/P1 e TPP, respectivamente, encontramos diferenças significativas entre PASc, PADc e PADp em detectar essas diferenças em P2/P1; a PADc e a PADp foram capazes de detectar essas diferenças no TPP (Tabela 3), e os mesmos parâmetros mantêm uma correlação significativa e moderada entre medidas de PA e as variáveis da PIC (Tabela 4).

Nosso estudo tem algumas limitações. É importante mencionar que esta é uma análise transversal da coorte, e estudos longitudinais são necessários para avaliar outras características em uma relação de causa e efeito. A força deste estudo é mostrar, pela primeira vez, dados de avaliações não invasivas da PA central e da PIC em pacientes hipertensos com doença crônica.

## Conclusão

Em conclusão, um aumento nos níveis de PASc e não nos níveis de PASp parece estar mais intimamente associado à HTIC. Em relação à PAD, tanto os valores centrais como os valores periféricos têm uma associação similar com HTIC. Esses resultados levantam a questão de qual seria a abordagem para avaliar a pressão arterial no contexto das doenças cerebrais.
