# Supplementary material for: Avaliação Não Invasiva da Pressão Arterial Central e da Forma de Onda Intracraniana em Pacientes Hipertensos: Um Estudo Transversal
Source: Arq Bras Cardiol. 2025 Apr 24;122(5):e20240778. [Article in Portuguese] doi: 10.36660/abc.20240778 (PMC12129485; doi:10.36660/abc.20240778)
Supplement: Translation

# Translation: Non-Invasive Central Blood Pressure and Intracranial Waveform Assessment in Hypertensive Patients: A Cross-Sectional Study

**DOI:** 10.36660/abc.20240778i

**Authors:** Sayuri Inuzuka, Mikaelle Costa Correia, Matheus Martins da Costa, Thiago Oliveira Costa, Priscila Valverde de Oliveria Vitorino, Polyana Vulcano de Toledo Piza, Gustavo Frigieri, Ana Luiza Lima Sousa, Antonio Coca, Weimar Kunz Sebba Barroso

**Affiliations:** 1 Brazil Unidade de Hipertensão Arterial – Universidade Federal de Goiás, Goiânia, GO – Brazil; 2 Brazil Pontifícia Universidade Católica de Goiás – Escola de Ciências Sociais e da Saúde, Goiânia, GO – Brazil; 3 Brazil Hospital Israelita Albert Einstein, Goiânia, GO – Brazil; 4 Brazil Brain4care, São Paulo, SP – Brazil; 5 Brazil Laboratório de investigação médica 62, Universidade de São Paulo, São Paulo, SP – Brazil; 6 Spain School of Health and Life Sciences. Universitat Abat Oliba CEU, CEU Universities, Barcelona – Spain; 7 Brazil Hospital das Clínicas da Universidade Federal de Goiás, EBSERH, Goiânia, GO – Brazil

**Keywords:** Stroke, Cerebrovascular Disorders, Cognitive Dysfunction, Hypertension, Intracranial Pressure

## Abstract

**Background:**

There is a strong association between hypertension and cerebrovascular disease, mainly with stroke and cognitive impairment. However, but the mechanistic of this relationship are not completely understood.

**Objective:**

To analyze the relationship of central, peripheral blood pressure (BP) and arterial stiffness, with intracranial pressure (ICP) in long-term chronic hypertensive patients.

**Methods:**

Adult individuals were consecutively included in the study from November 2022 to August 2023. The cut-off point identified to define intracranial hypertension (ICHT) by the wave peak (P2/P1) ratio was > 1.2, and the cut-off for time to peak (TTP) was > 0.25. The level of significance adopted in the statistical analysis was 5%.

**Results:**

A total of 145 patients (32 male, 113 female) with long-term hypertension (average of time since diagnoses 20 ± 12 years) were evaluated over a period of 10 months. The median age was 69.0 (61.8 – 75.7) years and median body mass index 29.0 (25.4 – 33.1) kg/m2. Median value of P2/P1 ratio for all cohort was 1.4 (1.2 – 1.5) and TTP 0.24 (0.21 – 0.29). The analysis was performed considering presence or not of ICHT, and parameters of central BP and pulse wave velocity. There was higher central systolic (SBP), diastolic blood pressure (DBP), and peripheral DBP among patients with ICHT based on the P2/P1 ratio.

**Conclusions:**

Central SBP levels are more linked to ICHT than office peripheral SBP measurements, while DBP measurements are similar, raising questions about the most suitable BP assessment method for hypertensive patients with cerebrovascular damage.

## Introduction

Hypertension (HT) remains as the leading cause of death around the globe, and brain diseases, such as stroke and cognitive decline as important health problems, affecting one in five Americans aged ≥ 65 years.^1,2^

There is a strong association between HT and cerebrovascular diseases, but the mechanistic bases of this relationship are not completely understood and further investigation on the issue is warranted. It is well established that blood pressure (BP) elevation may cause structural and functional damage in the arterial bed, but to at level this process begins and to what extent it can affect the brain remains to be elucidated.^2-5^

A recent study assessing non-invasive intracranial pressure (ICP) waveform in chronic hypertensive patients has found a 45.6% prevalence of intracranial hypertension (ICHT), raising some questions about the real protection capacity of cerebral autoregulation and vascular brain barriers in this population.^6-8^

Lately, the consequences of pulsatile arterial hemodynamics, including higher central BP and arterial stiffness have appeared as one of the important mechanisms that affect the function and structure of the brain. The presence of increased pulsatility in the small cerebral arteries as well as the brain endothelial cyclic mechanical stretch has the potential to produce amyloid deposits and reinforces the role, not only of peripheral BP, but also of central BP behavior in brain health.^9,10^

Hemodynamic autoregulation has the capacity to protect the brain tissue and vessels, but this protection may be altered when long-term hypertension modifies the capacity of brain vessels to adapt the flow to the increase in BP. We hypothesized that, after a long-term exposure to HT, the vascular protective mechanism of the brain is impaired. For this reason, we designed a cross- sectional study to explore the relationship of peripheral and central blood BP, and arterial stiffness, with ICP in long-term chronic hypertensive patients.

## Material and methods

### Selection of patients

A cohort of consecutive adult patients with hypertension seen in the Research Center for Cardiometabolic Diseases of the Hypertension Unit, Federal University of Goias, Brazil, from November 2022 to August 2023 were included in the cross-sectional analysis. The study was conducted in accordance with the resolution 466/2012 and was approved by the Ethic Committee number 70448823.1.0000.5078.

After a routine medical history and physical examination, the following parameters were recorded: age, sex, body mass index (BMI) in Kg/m^2^, office peripheral systolic (pSBP) and diastolic (pDBP) blood pressure in mmHg, central systolic (cSBP) and diastolic (cDBP) blood pressure in mmHg, pulse wave velocity (PWV) in m/sec, augmentation index (AIx) in %, previous history of cardiovascular (CV) risk factors and CV disease – myocardial infarction (MI), stroke, type 2 diabetes (DM2), and dyslipidemia, and the number and class of anti-hypertensive medications. All these variables were collected on the same day when the non-invasive ICP waveform was measured and were managed using the REDCap electronic data capture tool.

### Assessment of peripheral and central Blood pressure

The following parameters: pSBP, pDBP, cSBP, cDBP, PWV, and AIx were obtained using a validated oscillometric device, the Mobil-O-Graph® (IEM, Stolberg, Germany),^11,12^ based on triplicate measurements of PWV with C1 calibration (diastolic mean). The data were processed with the ARCSolver® algorithm (Austrian Institute of Technology, Vienna, Austria). The measurements were performed on the left arm, with the patient in a seated position with the legs uncrossed, feet flat on the floor, and the arm resting at heart level on a table. Patients were instructed to avoid alcohol consumption for 10 hours, refrain from caffeine intake, smoking, and exercise for three hours prior to the measurement, and rest for 10 minutes before the procedure. Three readings of central BP were obtained, and the average of the three measurements was calculated.^13,14^

### Assessment of intracranial pressure

A non-invasive ICP measurement was performed with patients in a lying position and monitored for seven minutes. The first and last minutes were discarded. The validated brain4care (b4c) sensor^8,15^was positioned on patient’s scalp, and the morphology of the waves was acquired through a strain sensor that could detect and monitor nanometric skull bone deformations during each cardiac cycle.

By the traditional invasive measurements, ICHT has been defined as sustained (> 5 min) IPC over 20 mmHg.^16^ Using the b4c non-invasive evaluation, the cut-off point identified to define ICHT by the P2/P1 ratio was ≥ 1.2, and the cut-off for time-to-peak (TTP) was ≥ 0.25 seconds. The P2/P1 ratio stands for the ratio between P1 amplitude (resulting from transmission of systolic cerebral blood flow) and P2 amplitude (associated with brain compliance to intracranial pressure), and TTP refers to the time in seconds from the beginning of waveform inscription until P1. The values of P2/P1 from 1.0 to 1.19 and the TTP values from 0.20 to 0.24 seconds were considered a grey zone of abnormal intracranial compliance but not ICHT^17-19^(Central Illustration).

### Statistical analysis

Data were recorded on the REDCap platform and analysed with Jamovi, version 2.3.28. Categorical variables were presented as frequencies and proportions. Quantitative variables were firstly analysed in terms of distribution, applying the Shapiro-Wilk test; these data were expressed as mean ± standard deviation (SD) or median and interquartile range, according to the normality of the data. To compare P2/P1 and TTP values between the categories of quantitative variables, the Mann-Whitney test was used for bivariate analyses, and, for correlations, the Spearman test was used. It was adopted as significant at p < 0,05.

## Results

A cohort of 145 patients with long-term essential HT was included in the study. The mean time since the hypertension diagnosis was 20.0 ± 12.0 years. Sociodemographic, anthropometric, and clinical variables are shown in Table 1. The comparison of P2/P1 and TTP values according to CV risk factors, and sociodemographic and anthropometric data did not find differences, except for TTP among males and females (Table 2).

**Table 1:** – Sample description according to sociodemographic characteristics, body mass index, comorbidities, number of anti-hypertensives, central blood pressure parameters and intracranial pressure parameters, n = 145

Variable	Mean ± SD or Median (p25 – p75%) or n (%)
**Age (years)**	69.0 (61.8 – 75.7)
**Sex**	
Female	113 (77.9%)
Male	32 (22.1%)
**Body Mass Index (n=136)**	29.0 (25.4 – 33.1)
**Comorbidities**	
Diabetes	74 (51.0%)
Dyslipidemia	129 (89.0%)
Myocardial Infarction	20 (13.8%)
Stroke	17 (11.7%)
**Number of anti-hypertensives**	
1	35 (24.1%)
2-3	43 (29.7%)
≥ 3	57 (39.3%)
**Blood Pressure Assessment**	
pSBP (mmHg)	125.0 (115.0 – 139.0)
pDBP (mmHg)	79.0 (72.0 – 86.0)
cSBP (mmHg)	115.0 (160.0 – 129.0)
cDBP (mmHg)	80.0 (73.0 – 87.0)
PWV (m/sec)	9.9 ± 1.9
Aix (%)	25.0 (16.0 – 30.0)
Vascular resistance (s*mmHg/ml)	1.4 (1.3 – 1.5)
**Intracranial Pressure Waveform**	
P2/P1	1.4 (1.2 – 1.5)
TTP	0.24 (0.21 – 0.29)

pSBP: peripheral systolic blood pressure; pDBP: peripheral diastolic blood pressure; cSBP: central systolic blood pressure; cDBP: central diastolic blood pressure; Aix: Augmentation index; PWV: pulse wave velocity; TTP: time to peak. Goiânia, 2023. Source: Author.

**Table 2:** – Comparison of P2/P1 and time to peak (TTP) across different clinical, sociodemographic and anthropometric parameters (n = 145)

Variable	P2/P1		TTP	
	Median (p25 – p75%)	p	Median (p25 – p75%)	p
**Age**		0.949		0.143
< 65 years	1.35 (1.23-1.52)		0.25 (0.23-0.29)	
≥ 65 years	1.37 (1.17-1.52)		0.24 (0.20-0.28)	
**Sex**		0.292		0.048
Female	1.37 (1.21-1.54)		0.25 (0.21-0.29)	
Male	1.33 (1.18-1.45)		0.23 (0.21-0.26)	
**Overweight (BMI>25)**		0.929		0.225
Yes	1.36 (1.19-1.52)		0.24 (0.22-0.29)	
No	1.36 (1.22-1.49)		0.23 (0.21-0.27)	
**Comorbidities**				
**Diabetes**		0.349		0.169
Yes	1.33 (1.20-1.49)		0.24 (0.21-0.27)	
No	1.39 (1.19-1.57)		0.25 (0.21-0.29)	
**Dyslipidemia**		0.334		0.614
Yes	1.35 (1.19-1.52)		0.24 (0.21-0.29)	
No	1.43 (1.23-1.64)		0.26 (0.20-0.28)	
**Myocardial infarction**		0.744		0.234
Yes	1.34 (1.14-1.57)		0.22 (0.20-0.28)	
No	1.36 (1.21-1.52)		0.25 (0.22-0.29)	
**Stroke**		0.890		0.993
Yes	1.29 (1.15-1.58)		0.24 (0.20-0.29)	
No	1.36 (1.20-1.52)		0.24 (0.21-0.28)	
**Number of anti-hypertensives**		0.855		0.481
1	1.37 (1.22-1.52)		0.25 (0.22-0.28)	
2	1.27 (1.18-1.47)		0.24 (0.21-0.28)	
≥ 3	1.40 (1.22-1.53)		0.25 (0.21-0.29)	

P2/P1 ratio: ratio between P1 amplitude (resulting from transmission of systolic cerebral blood flow) and P2 amplitude (associated with brain compliance to intracranial pressure). TTP: measurement in seconds of the beginning of waveform inscription until P1; TTP: time to peak; Mann-Whitney test. Goiânia, 2023. Source: Author.

According to the results of previous studies with P2/P1, we defined P2/P1 < 1.0 as normal, P2/P1 1.0 – 1.19 as intracranial compliance disturbance and P2/P1 ≥ 1.2 as ICHT.^17-19^ The association of ICHT with central BP and PWV values was also evaluated. Higher cSBP, cDBP, and pDBP values were found in patients with ICHT, identified by the P2/P1 ratio criterion. In ICHT patients, identified by adopting the TTP criterion (≥ 0.25 seconds), both central and peripheral DBPs were higher in these patients (Table 3). Table 4 shows the correlation analysis of ICP variables and central BP measurements.

**Table 3:** – Bivariate analysis of P2/P1 and time to peak (TTP) according to the central blood pressure and pulse wave velocity parameters

	P2/P1			TTP		
	< 1.2	≥ 1.2	p	< 0.25	≥ 0.25	p
pSBP	122.0 (112.0-133.0)	127.0 (116.0-139.0)	0.068	123.0 (115.0-136.0)	128.0 (115.5-140.5)	0.307
pDBP	75.0 (70.0-81.0)	80.0 (74.0-86.0)	0.030	77.0 (71.0-82.8)	80.0 (75.0-88.0)	0.032
cSBP	109.0 (103.0-117.0)	117.0 (107.8-131.0)	0.024	114.0 (106.0-123.0)	118.0 (107.0-131.0)	0.117
cDBP	76.0 (71.0-82,0)	81.0 (75.0-88.0)	0.037	78.5 (72.0-84.0)	81.0 (76.0-89.0)	0.039
PWV	10.1±2.0	9.8±1.9	0.438	10.1±2.0	9.7±1.8	0.149
Aix	25.0 (17.0-28.0)	25.0 (16.0-31.0)	0.572	25.0 (15.3-28.0)	26.0 (19.0-31.0)	0.190
VR	1.40 (1.30-1.50)	1,40 (1.30-1.53)	0.588	1.40 (1.30-1.54)	1.40 (1.30-1.50)	0.964

P2/P1 ratio: ratio between P1 amplitude (resulting from transmission of systolic cerebral blood flow) and P2 amplitude (associated with brain compliance to intracranial pressure). TTP: measurement in seconds of the beginning of waveform inscription until P1. pSBP: peripheral systolic blood pressure; pDBP: peripheral diastolic blood pressure; cSBP: central systolic blood pressure, cDBP: central diastolic blood pressure; PWV: pulse wave velocity; Aix: Augmentation index; VR: vascular resistance. Results obtained after use of the Mann-Whitney test. Source: Author.

**Table 4:** – Correlation analysis of P2/P1 and time to peak (TTP) with central blood pressure and pulse wave velocity assessments

	P2/P1		TTP	
	r	p	r	p
pSBP	0.16	0.053	0.09	0.299
pDBP	0.17	0.043	0.23	0.006
cSBP	0.19	0.020	0.14	0.095
cDBP	0.17	0.044	0.22	0.008
PWV	-0.02	0.767	-0.14	0.104
Aix	0.06	0.501	0.12	0.157
VR	-0.01	0.889	-0.06	0.509

P2/P1 ratio: ratio between P1 amplitude (resulting from transmission of systolic cerebral blood flow) and P2 amplitude (associated with brain compliance to intracranial pressure). TTP: measurement in seconds of the beginning of waveform inscription until P1. pSBP: peripheral systolic blood pressure; pDBP: peripheral diastolic blood pressure; cSBP: central systolic blood pressure, cDBP: central diastolic blood pressure; PWV: pulse wave velocity; Aix: Augmentation index; VR: vascular resistance Spearman correlation test. Source: Author.

## Discussion

The study cohort was composed of patients with chronic essential hypertension patients with a median age of 69.0 (61.8 – 75.7) years, a high prevalence of cardiometabolic risk factors such as DM2 (51.0%) and dyslipidemia (89.0%), and previous CV disease such as MI (13.8%) and stroke (11.7%) (table 1).

Although the strong association between HT and cerebrovascular disease is well established, particularly stroke and cognitive impairment, the pathophysiological mechanisms involving hypertension-induced brain damage are not well known. It seems that cerebral damage due to blood-brain barrier (BBB) and vascular autoregulation disorders occurs before neurodegeneration, and a better understanding of this link is of utmost importance.^20-24^

In a previous study,^6^ we found an unexpectedly high prevalence (45.6%) of ICHT in hypertensive patients, mainly in women, although no differences were observed between controlled and uncontrolled hypertensive patients using peripheral BP assessment. These findings have raised the hypothesis that after a long-term exposure to HT, the vascular protective mechanisms of the brain have been lost. However, another hypothesis to be considered is that peripheral BP measurements are not so accurate to detect these differences, and perhaps central BP measurements would have higher sensitivity. Noninvasive central assessments reflect BP values of large arteries such as aorta or carotids and, for this reason, are a stronger marker of CV morbidity and mortality than peripheral BP ^23,24^. Another important point is that central BP has a stronger association with target organ damage than peripheral BP.^25,26^ In this study, we found a median value of P2/P1 ratio of 1.4 (1.2 – 1.5), and a TTP median value of 0.24 (0.21 – 0.29) seconds in the study population. Table 1 shows values of peripheral and central BP values, and PWV analysis in this well-controlled high-risk hypertensive population featuring a high prevalence of CV risk factors and diseases as well as a median PWV close to 10 (9.9 ± 1.9) m/sec, which characterizes vascular damage according to previous studies and guidelines.^14,27-29^

When looking for differences in mean P2/P1 ratio and TTP in the sociodemographic, anthropometric, and clinical variables (Table 2), the only difference found was a longer TTP among females. Others authors, as well as our research group, have discussed that a long-term exposure to high BP could be responsible for important damage to neuroprotective mechanisms and a loss of capacity to maintain normal ICP values in this population, even when BP is well controlled.^3,6,9,30^It is important to mention that the incidence of stroke and dementia is higher among females, and the findings of a longer TTP, as well as a greater P2/P1 ratio among women in this study, support the hypothesis that the autoregulatory capacity of ICP and BBB permeability are differently affected in male and female.^9,31^

Numerous pathophysiological mechanisms have been associated with elevated BMI and ICHT, and obesity is recognized as a risk factor for the development of ICHT.^32,33^ Recent data suggest the association of ICHT with insulin resistance, DM2, and CV disease.^34,35^ However, we did not find any relationship of overweight, P2/P1 ratio or TTP with the presence of DM2 or dyslipidemia, perhaps due to the small study sample. Finally, in the analysis considering the cut-offs of 1.2 and 0.25 seconds for P2/P1 and TTP, respectively, we have found significant differences between cSBP, cDBP, and pDBP in detecting these differences concerning P2/P1; cDBP and pDBP were able to detect these differences concerning TTP (table 3), and the same parameters maintain a moderate and significant correlation between the BP measurements and ICP variables (table 4).

Our study has some limitations. It is important to note that it is a cross-sectional analysis of the cohort, and longitudinal studies are needed to evaluate other features in a cause-and-effect relationship. The strength of this study is to show for the first time data of non-invasive central BP and ICP assessments in hypertensive patients with chronic disease.

## Conclusion

In conclusion, an increase in cSBP rather than pSBP levels seems to be more closely associated with ICHT. Concerning DBP, both central and peripheral values have a similar association with ICHT. These findings raise the question of what approach is most appropriate for assessing blood pressure in the setting of brain disorders.
